# The Relationship between Shoe Fitting and Foot Health of Persons with Down Syndrome: A Case Control Study

**DOI:** 10.3390/ijerph15050983

**Published:** 2018-05-14

**Authors:** César Calvo-Lobo, Ana Ramos García, Marta Elena Losa Iglesias, Daniel López-López, David Rodríguez-Sanz, Carlos Romero-Morales, Ricardo Becerro-de-Bengoa-Vallejo

**Affiliations:** 1 Nursing and Physical Therapy Department, Institute of Biomedicine (IBIOMED), Faculty of Health Sciences, Universidad de León, Ponferrada, 24401 León, Spain; ccall@unileon.es; 2 Research, Health and Podiatry Unit, Department of Health Sciences, Faculty of Nursing and Podiatry, Universidade da Coruña, 15403 Ferrol, Spain; ana.ramos@udc.es; 3 Faculty of Health Sciences, Universidad Rey Juan Carlos, 28922 Alcorcón, Spain; marta.losa@urjc.es; 4 Physical therapy & Health Sciences Research Group Department, Faculty of Health, Exercise and Sport, European University of Madrid, Villaviciosa de Odón, 28670 Madrid, Spain; davidrodriguezsanz@gmail.com (D.R.-S.); carlos.romero@universidadeuropea.es (C.R.-M.); 5 School of Nursing, Physiotherapy and Podiatry, Universidad Complutense de Madrid, 28040 Madrid, Spain; ribebeva@ucm.es

**Keywords:** Down syndrome, foot, musculoskeletal disease, shoes

## Abstract

*Background*: Down syndrome is the most common chromosomal abnormality and a cause of intellectual disability. It is also associated with orthopaedic and musculoskeletal problems of the locomotive apparatus, especially of the feet. These problems are believed to have a harmful effect on health, social functioning, and mobility. In addition, these persons generally don’t have access to podiatric health services, even when their foot problems are well known, because of limited access to healthcare facilities. The goal of our research was to evaluate and compare the foot health status of study participants with and without Down syndrome and to determine whether inadequate footwear is being used with normalized reference values. *Methods*: A total of 105 participants with and without Down syndrome, with a mean age of 35.71 (SD = 12.93) years, were enrolled in the study. They self-reported demographic data and their clinical characteristic data were recorded. Measurements of their foot and shoe fitting were taken at all stages of the research process. Ninety-two percent of the participants with Down syndrome had foot problems. *Results:* Only 12 (24%) participants with Down syndrome used bilateral shoes that met the requirements of their feet compared to their controls (50 participants, 90.9% for the right foot; 46 participants, 83.6% for the left foot). Participants with Down syndrome presented statistically significant differences with respect to controls and wore incorrectly sized shoe. *Conclusions*: Evaluation of foot length and width may prevent development of foot deformities, as well as to improve general health.

## 1. Introduction

Down syndrome is the most common chromosomal abnormality and a cause of intellectual disability. Down syndrome has a live birth prevalence approaching 1 per 1000 births in developed countries [1,2].

Persons with Down syndrome have several concerns usually have learning disabilities [3], and often heart defects [4,5,6], craniofacial dysmorphia [7], gastrointestinal defects [8], childhood leukaemia [9,10], diabetes [11], obesity [12], musculoskeletal disorders [13] and orthopaedic problems [14]. In addition, more than 66% of Down syndrome patients have foot problems related to hypotonia and laxity of the muscles and ligaments [15], yet care of their feet is often neglected.

People with Down syndrome therefore are not getting sufficient access to healthcare [16,17] and need regular podiatric examinations and follow-up. The impacts of Down syndrome pose a significant burden to public and individual health, given the high incidence of inadequate footwear associated with metatarsus adductus [18], hallux valgus [19], metatarsus primus varus [20], hammertoes [19], pes planus [21,22], knee valgum [15], orthopaedic foot surgery [23], joint laxity [2], alterations of the lower limbs and associated fatigability, difficulties in walking [24], postural alterations [25], worse static balance [26] and foot pain [27].

Changes of the foot morphology occur during this period of life, resulting in increased foot width and length. Attention to the foot health of persona with Down syndrome has important benefits to their health, social functioning, and mobility [22,28]. Poorly fitting shoes and deformities of the foot, postural alterations, and other basic illnesses as factors should be taken into account when planning treatment and preventive care activities for the Down syndrome persons.

The goal of our research was to evaluate the foot conditions of persons with Down syndrome and to determine they were wearing suitable footwear. We hypothesized that foot health status in Down syndrome persons all favours the use of inadequate footwear in these persons with respect to controls.

## 2. Materials and Methods

### 2.1. Participants

This descriptive observational study was performed at a health centre in the city of Ferrol, in northwest Spain, from September 2015 to June 2016. A non-randomised and consecutive sampling method was used to select the 50 participants with Down syndrome.

Their parents or legal guardians provided informed consent for them to participate in the study. Inclusion criteria were Down syndrome with a low-to-medium intelligence quotient, no clinical sign of dementia, and no history of previous surgery or other significant orthopaedic treatments of the foot. Exclusion criteria were a prior history of foot trauma or foot surgery; existing use of foot orthoses or other orthotic inserts; or refusal of parents to sign the informed consent form [24,25,28].

### 2.2. Procedure

A single trained podiatry examiner collected measurements. Information on participants’ general health status and demographic characteristics such as sex, age, history of injury, hypotonia, height, and weight were also collected. Body mass index (BMI) was calculated by applying Quetelet’s equation (body weight in kilograms over body height in meters squared as follows: BMI = weight/height^2^) [29].

Participants then removed their footwear and hosiery. Their feet were examined for the following the protocol described by Concolino et al. [15]: general appearance, abnormalities of the toes, condition of the toenails, rotation of the feet, and presence of arches, foot type in the led podoscope device (Herbitas.com, Polígono Industrial, Foios, Valencia, Spain) and skin pathology [30].

To determine generalized ligamentous hyperlaxity (GLH), the modified Beighton 9-point scoring system was used [31]. This test is valid for diagnosis of GLH and showed high kappa values (intraobserver: 0.75; interobserver: 0.78) [32]. Five body areas of participants were examined: the fifth metacarpophalangeal joint, the elbows, the knees and the trunk. A positive result was recorded if a participant presented a cut-off of ≥4 hypermobile joints [33,34].

A validated Brannock Device-type measuring instrument was then used to record foot length and width and shoe fitting [35]. Each participant stood barefoot in a relaxed posture with the feet slightly apart and weight evenly distributed. The researcher helped participants to place their foot correctly in the device, with the heel located against the back of the heel cup, and measured the distance to the end of the longest toe (which was not necessarily the first toe). The same protocol was used for the other foot and external shoe fitting measurements that were recorded and compared.

This study was conducted according to the Strengthening the Reporting of Observational Studies in Epidemiology guidelines (STROBE) [36].

### 2.3. Ethics Considerations

This research was approved by the Research and Ethics Committee of the Universidade da Coruña, file number CE 18/2016. All the parents and/or legal guardians gave their informed consent before the participants were included in the study. The study adhered to the ethical standards for research on human beings based on the Declaration of Helsinki (World Medical Association) and the Convention of the Council of Europe on Human Rights and Biomedicine, as well as those based on the Universal Declaration of the UNESCO on the Human Genome and Human Rights and other appropriate national or institutional organisations.

### 2.4. Sample Size Calculation

The sample size was calculated with the software from Unidad de Epidemiología Clínica y Bioestadística, Complexo Hospitalario Universitario de A Coruña, Universidade da Coruña (available at http://www.fisterra.com/mbe/investiga/9muestras/9muestras2.asp) [37]. Considering the prevalence of 29.1 cases of Down syndrome per 10,000 persons in the northwestern region of Spain and the total population of 69,452 persons (available on http://www.ine.es/ jaxiT3/Datos.htm?t=2868) in the city of Ferrol [38], the sample size calculation for an α level of 5% (confidence interval, α-1 = 95%), an error β of 20% with a power analysis of 80%, a proportion of 5% and a precision of ±7%, produced a minimum of n = 37 cases. Furthermore, assuming information loss of 15%, at least n = 44 cases must be studied. Finally, 50 people with Down syndrome and 55 without were included in the study [39,40].

### 2.5. Statistical Analysis

Statistical analyses were performed using IBM SPSS statistical software (Version 19.0, IBM Corp., Armonk, NY, USA). The Kolmogorov-Smirnov test was used to test the normality of the data. The results of these test indicated that the data were normally distributed and that parametric statistical tests were most appropriate. Independent Student t tests were performed to determine whether there were statistically significant differences in height, weight, age and BMI between the two groups. Categorical variables are shown as absolute values and percentages, while the quantitative variables described are the mean, standard deviation (SD), and maximum and minimum values. A Fisher’s Exact Test compared the qualitative variables and the independent Student’s *t*-test was used to compare the means of the groups.

## 3. Results 

### Sample Characteristics

A total of 105 participants with and without Down syndrome completed all the stages of the research process. Fifty participants had Down syndrome (52%) and 55 did not (48%). The ages ranged (minimum–maximum) from 15 to 63 years, with a mean of age of 35.71 (SD = 12.93) years.

Table 1 shows the clinical and sociodemographic characteristics of the participants. Most participants were overweight (BMI = 26.39 kg/m^2^).

Generally, 92% (n = 46) of participants with Down syndrome stated that they had foot problems; subsequent physical examinations revealed that all 46 had flatfoot, 24 (48%) had metatarsus primus adductus, 26 (52%) had hypermobile first ray, 26 (52%) had nail lesions, 40 (80%) had hyperkeratotic lesions, and 6 (12%) had fungal infections. Additionally, 73% of the participants had hypotonia (n = 45; 90%), or muscle and ligament laxity (n = 39; 78%).

Foot and shoe fitting were measured using a Brannock device; 76% participants were found to be wearing inadequate footwear. Table 2 shows that only 12 participants (24%) wore shoes that met the needs and requirements of their feet, with regards to width or length when compared to their controls. Among the controls, 46 persons (83.63%) wore correct footwear. The significant differences are shown in Table 3.

Also, only eight (16%) participants with Down syndrome wore correct footwear for their right foot compared to their controls. Almost all the control participants (50 participants; 90.9%) had correct footwear for their right foot. Similar results were found for the left foot. Only eight (16%) participants with Down syndrome wore adequate left shoe fitting compared to their control group (46 participants; 83.6%). Participants with Down syndrome presented statistically significant differences with respect to controls regarding incorrectly sized shoes, as shown in Table 4 and Table 5.

## 4. Discussion

Footwear that is either therapeutic and custom-made or off-the-shelf is recommended for people with disorders, such as flat feet. Such footwear can reduce foot pain, improve foot health, and increase general mobility [41].

People with Down syndrome generally don’t have access to podiatric health services even when their foot problems are well known. Government-funded health services in Spain do not cover podiatric care services. Nor does private insurance. Therefore, these patients cannot request podiatric advice and services.

We performed a cross-sectional study to find out if information about the relationship between shoe fitting and foot health among persons with Down syndrome was adequate to promote foot health and avoid foot pathology. Most studies on this issue concern detection of foot problems in children with Down syndrome [15,42,43]. However, to our knowledge, there are no studies that demonstrate that careful podiatric examination of adults with Down syndrome shows an elevated incidence of foot disorders.

Our research demonstrates that careful podiatric examination of persons with Down syndrome shows an elevated incidence of foot disorders. Most of the anomalies were secondary to hypotonia and laxity of the muscles and ligaments. This finding is consistent with the findings of other studies that investigated foot problems, and suggests that the most critical problems are based on uncommon orthopaedic abnormalities [14,25].

There are significant changes in the structure and function of the foot with age, which contribute to altered plantar loading patterns during gait. As people age, they exhibit flatter or more pronated feet with a higher incidence of hallux valgus, toe deformities, and toe muscles’ plantar flexor weakness [44].

Therefore, with age, the shoe should be changed to avoid foot injuries or aggravation of flat feet symptoms. This work has highlighted the need for good footwear and for better knowledge of footwear among patients’ caretakers and practitioners.

This topic has received little attention in persons with Down syndrome due to we did not find literature about, but several studies have discussed the importance of footwear in other population, emphasizing that a poorly fitting shoe was linked to increased impairment and foot pain [38,45,46,47,48,49].

Additionally, to our knowledge, this is the first study to report that Down syndrome persons wore incorrectly-sized shoes. Our results are consistent with those of an earlier study that showed a significant mismatch between foot and shoe fitting among Special Olympics athletes [48]. That finding further highlights what can appear to be obvious: that assessing the feet of Down syndrome persons must include an assessment of their footwear.

Furthermore, the current study was observational and we found that persons without Down syndrome wore proper shoes. Perhaps this was because they chose their footwear on their own. Relatives of persons with Down syndrome should be instructed how to choose a properly fitting shoe. Future studies need to evaluate cause and effect before any definitive conclusions can be made about the relationship between footwear, foot type, foot pathologies and associated comfort or pain in persons with Down syndrome. Their foot problems deserve it.

Indeed, future clinical trials with novel interventions like parents’ education programmes or the use of the Brannock device-type measuring instruments may improve the fitting of shoes according to the foot length and width of persons with Down syndrome [36,49].

This study has some limitations. First, this research was performed at an outpatient health care clinic with a relatively small number of participants. Second, a larger and more diverse sample size (including individuals with Down syndrome from various countries) would strengthen the study and identify more mechanisms involved. Third, only one evaluator analyzed the participants’ feet. Lastly, futures studies should have at least two blinded evaluators. One evaluator would conduct feet examinations and other would evaluate footwear convenience by comparing it to blinded recorded data. A blind control group without Down syndrome should also be compared to with Down syndrome to strengthen the results. Fourth, the groups are statistically different in age, height and BMI where similar groups could be more adequate for future studies. Finally, further research on this trend is needed to determine the various foot disorders and how physicians’ therapeutic interventions could improve the health of Down syndrome persons.

Moreover, the healthcare system in Spain and most countries in Europe is based on socialised medicine. However, while medical care is free citizens, it does not cover many supplies, such as orthotics and therapeutic shoes. Even private health insurances in most European countries does not cover them. Physicians are necessary to prescribe an expensive custom shoe that, in our clinical experience, few of this population can afford.

## 5. Conclusions

This study provides evidence that most participants with Down syndrome were wearing an incorrectly sized shoe, and that evaluation of foot length and width are an important component to prevent the appearance of medical conditions and/or foot deformities, as well as to improve general health.

## Figures and Tables

**Table 1:** Socio-demographic and clinical characteristics of the sample population.

Demographic Characteristics	Total Group Mean of SD Range n = 105	With DS Mean of SD Range n = 50	Without DS Mean of SD Range n = 55	*p* Value
Age, years	35.71	25.58	44.92	0.001
	(SD = 12.93)	(SD = 8.16)	(SD = 8.95)	
	(15–63)	(15–44)	(27–63)	
Weight (kg)	66.96	66.81	67.10	0.923
	(SD = 15.56)	(SD = 18.37)	(SD = 12.65)	
	(23–101.2)	(23–101.2)	(43–95)	
Height (cm)	158.64	149.70	166.60	0.001
	(SD = 15.25)	(SD = 16.20)	(SD = 8.41)	
	(100–187)	(109–167)	(152–187)	
BMI (kg/m^2^)	26.39	28.97	24.04	0.001
	(SD = 4.40)	(SD = 3.64)	(SD = 3.67)	
	(15.79–37.20)	(22.80–37.20)	(15.79–34.06)	

Abbreviations: BMI, body mass index; DS, Down syndrome; SD, standard deviation. In all the analyses, *p* < 0.05 was considered statistically significant.

**Table 2:** Foot and shoe measurements in participants with DS (both feet, standing position).

Standing Position	Excessive Shoe Width	Correct Shoe Width	Insufficient Shoe Width	Total	*p* Value
Right Foot					
Shoe fitting too big	1 (2.0%)	0 (0.0%)	12 (24%)		0.001
Correct shoe fitting	0 (0.0%)	8 (16.0%)	4 (8.0%)		
Shoe fitting too small	1 (2.0%)	4 (8.0%)	20 (40.0%)		
Total	2 (4.0%)	12 (24%)	36 (72%)	50(100%)	
Left Foot					
Shoe fitting too big	1 (2.0%)	0 (0.0%)	12 (24%)		0.001
Correct shoe fitting	0 (0.0%)	8 (16.0%)	4 (8.0%)		
Shoe fitting too small	1 (2.0%)	4 (8.0%)	20 (40.0%)		
Total	2 (4.0%)	12 (24%)	36 (72%)	50(100%)	

Abbreviations: DS, Down syndrome. In all the analyses, *p* < 0.05 was considered statistically significant.

**Table 3:** Foot and shoe measurements in participants without DS (both feet, standing position).

Standing Position	Excessive Shoe Width	Correct Shoe Width	Insufficient Shoe Width	Total	*p* Value
Right Foot					
Shoe fitting too big	0 (0.0%)	0 (0.0%)	0 (0.0%)		0.001
Correct shoe fitting	0 (0.0%)	50 (90.9%)	0 (0.0%)		
Shoe fitting too small	0 (0.0%)	0 (0.0%)	5 (9.1%)		
Total	0 (0.0%)	50 (90.9%)	5 (9.1%)	55 (100%)	
Left Foot					
Shoe fitting too big	0 (0.0%)	0 (0.0%)	0 (0.0%)		0.001
Correct shoe fitting	0 (0.0%)	46 (83.63%)	0 (0.0%)		
Shoe fitting too small	0 (0.0%)	0 (0.0%)	9 (16.3%)		
Total	0 (0.0%)	46 (83.6%)	9 (16.3%)	55(100%)	

Abbreviations: DS, Down syndrome. In all the analyses, *p* < 0.05 was considered statistically significant.

**Table 4:** Difference of right foot and shoe measurements in participants with and without DS (both feet, standing position).

Shoe Measurement in Standing Position	With DS n (%)	Without DS n (%)	*p* Value
Size too big and Excessive width	1 (2.0%)	0 (0.0%)	0.001
Size too big and Correct width	0 (0.0%)	0 (0.0%)	
Size too big and Insufficient width	12 (24%)	0 (0.0%)	
Correct shoe fitting and Excessive width	0 (0.0%)	0 (0.0%)	
Correct shoe fitting and Correct width	8 (16.0%)	50 (90.9%)	
Correct shoe fitting and Insufficient width	4 (8.0%)	0 (0.0%)	
Shoe fitting too small and Excessive width	1 (2.0%)	0 (0.0%)	
Shoe fitting too small and Correct width	4 (8.0%)	0 (0.0%)	
Shoe fitting too small and Insufficient width	20 (40.0%)	5 (9.1%)	
Total	50 (100%)	55 (100%)	

Abbreviations: DS, Down syndrome. In all the analyses, *p* < 0.05 was considered statistically significant.

**Table 5:** Difference of left foot and shoe measurements in participants with and without DS (both feet, standing position).

Shoe Measurement in Standing Position	With DS n (%)	Without DS n (%)	*p* Value
Size too big and Excessive width	1 (2.0%)	0 (0.0%)	0.001
Size too big and Correct width	0 (0.0%)	0 (0.0%)	
Size too big and Insufficient width	12 (24.0%)	0 (0.0%)	
Correct shoe fitting and Excessive width	0 (0.0%)	0 (0.0%)	
Correct shoe fitting and Correct width	8 (16%)	46 (83.6%)	
Correct shoe fitting and Insufficient width	4 (8.0%)	0 (0.0%)	
Shoe fitting too small and Excessive width	1 (2.0%)	0 (0.0%)	
Shoe fitting too small and Correct width	4 (8.0%)	0 (0.0%)	
Shoe fitting too small and Insufficient width	20 (40%)	9 (16.4%)	
Total	50 (100%)	55 (100%)	

Abbreviations: DS, Down syndrome. In all the analyses, *p* < 0.05 was considered statistically significant.

## References

[1] Thomas K., Girdler S., Bourke J., Deshpande A., Bathgate K., Fehr S., Leonard H. (2010). Chapter Three—Overview of Health Issues in School-aged Children with Down Syndrome. Int. Rev. Res. Ment. Retard..

[2] Amirfeyz R., Aspros D., Gargan M. (2006). Down syndrome. Curr. Orthop..

[3] Leonard S., Bower C., Petterson B., Leonard H. (1999). Medical aspects of school-aged children with Down syndrome. Dev. Med. Child. Neurol..

[4] Cleves M.A., Hobbs C.A., Cleves P.A., Tilford J.M., Bird T.M., Robbins J.M. (2007). Congenital defects among liveborn infants with Down syndrome. Birth Defects Res. A Clin. Mol. Teratol..

[5] Frid C., Annerén G., Rasmussen F., Sundelin C., Drott P. (2002). Utilization of medical care among children with Down’s syndrome. J. Intellect. Disabil. Res..

[6] Irving C.A., Chaudhari M.P. (2012). Cardiovascular abnormalities in Down’s syndrome: Spectrum, management and survival over 22 years. Arch. Dis. Child..

[7] Wiseman F.K., Alford K.A., Tybulewicz V.L.J., Fisher E.M.C. (2009). Down syndrome—Recent progress and future prospects. Hum. Mol. Genet..

[8] Van Allen M.I., Fung J., Jurenka S.B. (1999). Health care concerns and guidelines for adults with Down syndrome. Am. J. Med. Genet..

[9] Goldacre M.J., Wotton C.J., Seagroatt V., Yeates D. (2004). Cancers and immune related diseases associated with Down’s syndrome: A record linkage study. Arch. Dis. Child..

[10] Sullivan S.G., Hussain R., Glasson E.J., Bittles A.H. (2007). The profile and incidence of cancer in Down syndrome. J. Intellect. Disabil. Res..

[11] Van Goor J.C., Massa G.G., Hirasing R. (1997). Increased incidence and prevalence of diabetes mellitus in Down’s syndrome. Arch. Dis. Child..

[12] Van Schrojenstein Lantman-de Valk H.M., Haveman M.J., Crebolder H.F. (1996). Comorbidity in people with Down’s syndrome: A criteria-based analysis. J. Intellect. Disabil. Res..

[13] Hresko M.T., McCarthy J.C., Goldberg M.J. (1993). Hip disease in adults with Down syndrome. J. Bone Jt. Surg. Br..

[14] Diamond L.S., Lynne D., Sigman B. (1981). Orthopedic disorders in patients with Down’s syndrome. Orthop. Clin. N. Am..

[15] Concolino D., Pasquzzi A., Capalbo G., Sinopoli S., Strisciuglio P. (2006). Early detection of podiatric anomalies in children with Down syndrome. Acta Paediatr..

[16] Geelhoed E.A., Bebbington A., Bower C., Deshpande A., Leonard H. (2011). Direct health care costs of children and adolescents with Down syndrome. J. Pediatr..

[17] Michael J. (2009). Healthcare for People with Disabilities. https://www.healthcare.gov/people-with-disabilities/coverage-options/.

[18] Merrick J., Ezra E., Josef B., Hendel D., Steinberg D.M., Wientroub S. (2000). Musculoskeletal problems in Down Syndrome European Paediatric Orthopaedic Society Survey: The Israeli sample. J. Pediatr. Orthop. B.

[19] Roizen N.J., Patterson D., Gupta S., Hunt L., Lovell D., Levin B. (2003). Down’s syndrome. Lancet.

[20] Yam W.K., Tse P.W., Yu C.M., Chow C.B., But W.M., Li K.Y., Lee L.P., Fung E.L., Mak P.P., Lau J.T. (2008). Medical issues among children and teenagers with Down syndrome in Hong Kong. Down Syndr. Res. Pract..

[21] Pau M., Galli M., Crivellini M., Albertini G. (2012). Foot–ground interaction during upright standing in children with Down syndrome. Res. Dev. Disabil..

[22] Galli M., Rigoldi C., Brunner R., Virji-Babul N., Giorgio A. (2008). Joint stiffness and gait pattern evaluation in children with Down syndrome. Gait Posture.

[23] Cristofaro R.L. (1986). Orthopaedic abnormalities in an adult population with Down’s syndrome. Orhop Trans..

[24] Smith B.A., Ulrich B.D. (2008). Early onset of stabilizing strategies for gait and obstacles: Older adults with Down syndrome. Gait Posture.

[25] Pikora T.J., Bourke J., Bathgate K., Foley K.-R., Lennox N., Leonard H. (2014). Health conditions and their impact among adolescents and young adults with Down syndrome. PLoS ONE.

[26] Villarroya M.A., González-Agüero A., Moros-García T., de la Flor Marín M., Moreno L.A., Casajús J.A. (2012). Static standing balance in adolescents with Down syndrome. Res. Dev. Disabil..

[27] Benoit E.P. (1965). Podiatry and Mental retardation. The podiatrist’s. J. Am. Podiatry Assoc..

[28] Courtenay K., Murray A. (2015). Foot Health and Mobility in People With Intellectual Disabilities. J. Policy Pract. Intellect. Disabil..

[29] Garrow J.S., Webster J. (1985). Quetelet’s index (W/H2) as a measure of fatness. Int. J. Obes..

[30] Welton E.A. (1992). The Harris and Beath footprint: Interpretation and clinical value. Foot Ankle.

[31] Beighton P., Grahame R., Bird H. (1999). Clinical Features of Hypermobility: Locomotor System and Extra-articular. Hypermobility of Joints.

[32] Juul-Kristensen B., Røgind H., Jensen D.V., Remvig L. (2007). Inter-examiner reproducibility of tests and criteria for generalized joint hypermobility and benign joint hypermobility syndrome. Rheumatology.

[33] Seçkin Ü., Tur B.S., Yılmaz Ö., Yağcı İ., Bodur H., Arasıl T. (2005). The prevalence of joint hypermobility among high school students. Rheumatol. Int..

[34] Grahame R., Hakim A.J. (2008). Hypermobility. Curr. Opin. Rheumatol..

[35] Harrison S.J., Cochrane L., Abboud R.J., Leese G.P. (2007). Do patients with diabetes wear shoes of the correct size?. Int. J. Clin. Pract..

[36] White R.G., Hakim A.J., Salganik M.J., Spiller M.W., Johnston L.G., Kerr L., Kendall C., Drake A., Wilson D., Orroth K. (2015). Strengthening the Reporting of Observational Studies in Epidemiology for respondent-driven sampling studies: “STROBE-RDS” statement. J. Clin. Epidemiol..

[37] Fernández P. (1996). Investigación: Determinación del tamaño muestral Determinación del tamaño muestral. Cad. Aten. Primaria.

[38] Mosquera Tenreiro C., Ariza Hevia F., Rodríguez Dehli C., Fernández Toral J., García López E., Riaño Galán I. (2009). Frecuencia del síndrome de Down en Asturias y tendencia temporal, 1990–2004. Med. Clin..

[39] Williams A.E., Nester C.J., Ravey M.I. (2007). Rheumatoid arthritis patients’ experiences of wearing therapeutic footwear—A qualitative investigation. BMC Musculoskelet. Disord..

[40] Prasher V.P., Robinson L., Krishnan V.H., Chung M.C. (1995). Podiatric disorders among children with Down syndrome and learning disability. Dev. Med. Child. Neurol..

[41] Gutiérrez-Vilahú L., Massó-Ortigosa N., Rey-Abella F., Costa-Tutusaus L., Guerra-Balic M. (2015). Comparative study of plantar footprints in youth with Down syndrome. Int. Med. Rev. Down Syndr..

[42] Scott G., Menz H.B., Newcombe L. (2007). Age-related differences in foot structure and function. Gait Posture.

[43] Ikpeze T.C., Omar A., Elfar J.H. (2015). Evaluating Problems With Footwear in the Geriatric Population. Geriatr. Orthop. Surg. Rehabil..

[44] Dufour A.B., Broe K.E., Nguyen U.-S.D., Gagnon D.R., Hillstrom H.J., Walker A.H., Kivell E., Hannan M.T. (2009). Foot Pain: Is Current or Past Shoewear a Factor?. Arthritis Rheum..

[45] Riskowski J., Dufour A.B., Hannan M.T. (2011). Arthritis, foot pain and shoe wear: Current musculoskeletal research on feet. Curr. Opin. Rheumatol..

[46] Brenton-Rule A., Hendry G.J., Barr G., Rome K. (2014). An evaluation of seasonal variations in footwear worn by adults with inflammatory arthritis: A cross-sectional observational study using a web-based survey. J. Foot Ankle Res..

[47] Silvester R.N., Williams A.E., Dalbeth N., Rome K. (2010). “Choosing shoes”: A preliminary study into the challenges facing clinicians in assessing footwear for rheumatoid patients. J. Foot Ankle Res..

[48] Jenkins D.W., Cooper K., O’Connor R., Watanabe L. (2012). Foot-to-shoe mismatch and rates of referral in Special Olympics athletes. J. Am. Podiatr. Med. Assoc..

[49] Leonard H., Foley K.-R., Pikora T., Bourke J., Wong K., McPherson L., Lennox N., Downs J. (2016). Transition to adulthood for young people with intellectual disability: The experiences of their families. Eur. Child. Adolesc. Psychiatry.

